# Supplementary material for: Synergistic Inhibitory Effect of Polymyxin B in Combination with Ceftazidime against Robust Biofilm Formed by Acinetobacter baumannii with Genetic Deficiency in AbaI/AbaR Quorum Sensing
Source: Microbiol Spectr. 2022 Feb 23;10(1):e01768-21. doi: 10.1128/spectrum.01768-21 (PMC8865539; doi:10.1128/spectrum.01768-21)
Supplement: SUPPLEMENTAL FILE 1 — Supplemental material. Download SPECTRUM01768-21_Supp_1_seq9.pdf, PDF file, 0.8 MB [file spectrum01768-21_supp_1_seq9.pdf]

Table S1 Source information of *Acinetobacter baumannii* isolates

Isolates	Source of the patients	Source of samples	ERIC types
HF1	Respiratory and Critical Illness	Sputum	I
HF2	Traumatic Orthopedics	Wound	II
HF3	Traumatic Orthopedics	Wound	III
HF4	Urinary Surgery	Sputum	II
HF5	Intensive Care Unit	Sputum	IV
HF6	Intensive Care Unit	Sputum	II
HF7	Rheumatology and Immunology Department	Sputum	V
HF8	Neurosurgery Department	Sputum	II
HF9	Intensive Care Unit	Sputum	II
HF10	Emergency Internal Medicine	Urine	IV
HF11	Burn Unit	Wound	VI
HF12	Respiratory and Critical Illness	Sputum	IV
HF13	Intensive Care Unit	Sputum	III
HF14	Pediatric Intensive Care Unit	Sputum	V
HF15	Kidney Internal Medicine	Urine	V
HF16	Endocrine Department	Wound	V
HF17	Emergency Internal Medicine	Sputum	II
HF18	Department of Rehabilitation Sports Medicine	Urine	II
HF19	Burn Unit	Wound	V
HF20	Intensive Care Unit	Sputum	III
HF21	Respiratory and Critical Illness	Sputum	I
HF22	Intensive Care Unit	Sputum	III
HF23	Intensive Care Unit	Sputum	II
HF24	Intensive Care Unit	Cerebrospinal fluid	II
HF25	Intensive Care Unit	Sputum	II
HF26	Emergency Surgery	Wound	II
HF27	Intensive Care Unit	Sputum	III
HF28	Respiratory and Critical Illness	Sputum	V
HF29	Neurosurgery Department	Sputum	III
HF30	Intensive Care Unit	Sputum	III
HF31	Intensive Care Unit	Sputum	III
HF32	Intensive Care Unit	Sputum	VII
HF33	Intensive Care Unit	Sputum	V
HF34	Respiratory and Critical Illness	Sputum	II
HF35	Traumatic Orthopedics	Wound	II
HF36	Intensive Care Unit	Sputum	II
HF37	Intensive Care Unit	Sputum	II
HF38	Intensive Care Unit	Sputum	II
HF39	Intensive Care Unit	Sputum	III
HF40	Oncology Radiotherapy Department	Sputum	IV
HF41	Burn Unit	Wound	II

table to be continued.

Isolates	Source of the patients	Source of samples	ERIC types
HF42	Emergency Internal Medicine	Sputum	II
HF43	Hepatopancreatobiliary Surgery	Sputum	IV
HF44	Intensive Care Unit	Sputum	II
HF45	Department of Rehabilitation Sports Medicine	Sputum	II
HF46	Intensive Care Unit	Bile	II
HF47	Emergency Internal Medicine	Sputum	V
HF48	Dermatology and Venereology	Wound	IV
HF49	Respiratory and Critical Illness	Sputum	VIII
HF50	Intensive Care Unit	Sputum	III
HF51	Department of Cardiology	Sputum	IV
HF52	Intensive Care Unit	Sputum	II
HF53	Intensive Care Unit	Sputum	II
HF54	Intensive Care Unit	Sputum	II
HF55	Neurology Department	Urine	IV
HF56	Intensive Care Unit	Wound	II
HF57	Department of Cardiology	Sputum	IV
HF58	Burn Unit	Wound	II
HF59	Intensive Care Unit	Sputum	III
HF60	Intensive Care Unit	Sputum	III
HF61	Burn Unit	Wound	II
HF62	Respiratory and Critical Illness	Sputum	II
HF63	Intensive Care Unit	Sputum	II
HF64	Intensive Care Unit	Sputum	II
HF65	Intensive Care Unit	Venous catheter	II
HF66	Burn Unit	Wound	II
HF67	Traumatic Orthopedics	Wound	II
HF68	Cardiac Macrovascular Surgery	Blood	II
HF69	Emergency Surgery	Subcutaneous transudation	II
HF70	Intensive Care Unit	Sputum	II
HF71	Neurology Department	Sputum	II
HF72	Burn Unit	Wound	II
HF73	Intensive Care Unit	Sputum	II
HF74	Respiratory and Critical Illness	Sputum	II
HF75	Urinary Surgery	Blood	IV
HF76	Urinary Surgery	Urine	V
HF77	Respiratory and Critical Illness	Sputum	II
HF78	Respiratory and Critical Illness	Sputum	V
HF79	Intensive Care Unit	Sputum	II
HF80	Kidney Internal Medicine	Trachea cannula	II
HF81	Intensive Care Unit	Sputum	II
HF82	Intensive Care Unit	Sputum	II

table to be continued.

Isolates	Source of the patients	Source of samples	ERIC types
HF83	Emergency Surgery	Wound	II
HF84	Neurosurgery Department	Sputum	II
HF85	Burn Unit	Wound	II
HF86	Intensive Care Unit	Sputum	IV
HF87	Intensive Care Unit	Sputum	III
HF88	Emergency Surgery	Sputum	III
HF89	Intensive Care Unit	Nasopharynx	III
HF90	Intensive Care Unit	Sputum	II
HF91	Intensive Care Unit	Sputum	II
HF92	Intensive Care Unit	Sputum	II
HF93	Intensive Care Unit	Wound	II
HF94	Department of Rehabilitation Sports Medicine	Sputum	III
HF95	Intensive Care Unit	Sputum	V
HF96	Department of Infectious Diseases	Sputum	III
HF97	Intensive Care Unit	Sputum	IX
HF98	Burn Unit	Wound	III
HF99	Department of Digestion Internal Medicine	Sputum	V
HF100	Department of Neurology	Sputum	V
HF101	Department of Rehabilitation Sports Medicine	Sputum	II
HF102	Intensive Care Unit	Sputum	III
HF103	Emergency Surgery	Subcutaneous transudation	III

Table S2 The primers used for (RT)- PCR amplification

Target gene	Molecular function	Primer pair[†]	Direction[*]: sequence (5' - 3')	Amplicon size	Reference
adeA	a periplasmic membrane fusion protein	1	F: GGCGTATTGGGCAATCTTTTGT<br>R: GTCACCGACTTTCAAGCCTTTG	524	(1)
adeB	an inner membrane transporter	1	F: CGGAAGGCATGGAGTTTAG<br>R:TACTGCCGCCAATACCAG	404	this study
		2	F: CTTGCATTTACGTGTGGTGT<br>R: GCTTTTCTACTGCACCCAAA	169	(2)
adeC	an outer membrane channel	1	F: GACAATCGTATCTCGTGGACTC<br>R: AGCAATTTTCTGGTCAGTTTCC	1331	(1)
adeS	a sensor kinase of AdeABC transcription	1	F: GTGGACGTTAGGTCAAGTTCTG<br>R: TGTTATCTTTTGCGGCTGTATT	531	(1)
adeR	a response regulator of AdeABC transcription	1	F: TCACATGGCTATCTACGGTTGG<br>R: TGAAGGCATGAGTGTTATTCGG	533	(1)
adeF	a periplasmic membrane fusion protein	1	F: GGTGTCGACCAAGATAAACG<br>R: GTGAATTTGGCATAGGAACG	208	(3)
adeG	an inner membrane transporter	1	F: GGTTCTGGTGACTACGCAATG<br>R: CCGCGATATAGGCATCTTG	1471	this study
		2	F: GCCTGGTGTAGAAAGTGCTG<br>R: CCGCGATATAGGCATCTTG	191	this study
adeH	an outer membrane channel	1	F: CGATCAGCAAATTCAGGCTC<br>R: GCTTGCAATGATTTGGTTAC	181	this study
adeL	a transcriptional regulator of AdeFGH	1	F: GAAATCGGCATCGGTGCTG<br>R: GACGGTGATGCTTATATCGACT	140	this study
adeI	a periplasmic membrane fusion protein	1	F: CAACAAGTATCGCGCTTG<br>R: CTTAGTTGTTGACGTAAACGC	661	this study
adeJ	an inner membrane transporter	1	F: GCGGTCATTAATATCTTTGGC<br>R: CGTTAATGTATGAGCCACCC	1144	this study
		2	F: GGTCATTAATATCTTTGGC<br>R: GGTACGAATACCGCTGTCA	222	(2)
adeK	an outer membrane channel	1	F: CAAGTTGCTCAAGCATGG<br>R: CTAGATGGTTGGTGTACCAC	917	this study
adeN	a transcriptional regulator of AdeIJK	1	F: GAGCTAGGCATGGATTCTG<br>R: CACGTGCTGAATCTTCAATC	1387	this study
abaI	autoinducer synthase	1	F: AATGCCTATTCCCTGCTCAC<br>R: ATTGCTTCTTGCAGAATTGC	132	(4)
		2	F: AATGCCTATTCCCTGCTCAC<br>R: ATTGCTTCTTGCAGAATTGC	132	(4)
abaR	Quorum sensing receptor	1	F: ACCTCTTGTTTGGTCGAG<br>R: TCCTCGGGTCCCAATAA	127	(5)
		2	F: ACCTCTTGTTTGGTCGAG<br>R: TCCTCGGGTCCCAATAA	127	(5)
IS*Aba1*	Insertion sequence	1	F: AAGTAACACCAACAGAAGCT<br>R: TCTCTAAAATGGAACAAGTC	850	this study
bla$_{OXA-23}$	OXA-23 carbapenemase	1	F: TTTCTATTSATCTGGTGTTTA<br>R: TTAGAGGTTTCTGTCAAGCTC	865	(6)
		2	F: TTTCTATTSATCTGGTGTTTA<br>R: TGTTTGAATAACCAGCAC	194	this study
rpoB	Reference gene	2	F: ATGCCGCCTGAAAAAGTAAC<br>R: TCCGCACGTAAAGTAGGAAC	154	(4)

[†]1, primers used for gene detection; 2, primers used for transcriptional expression of gene. [*] F, forward; R, reverse.

FIG. S1

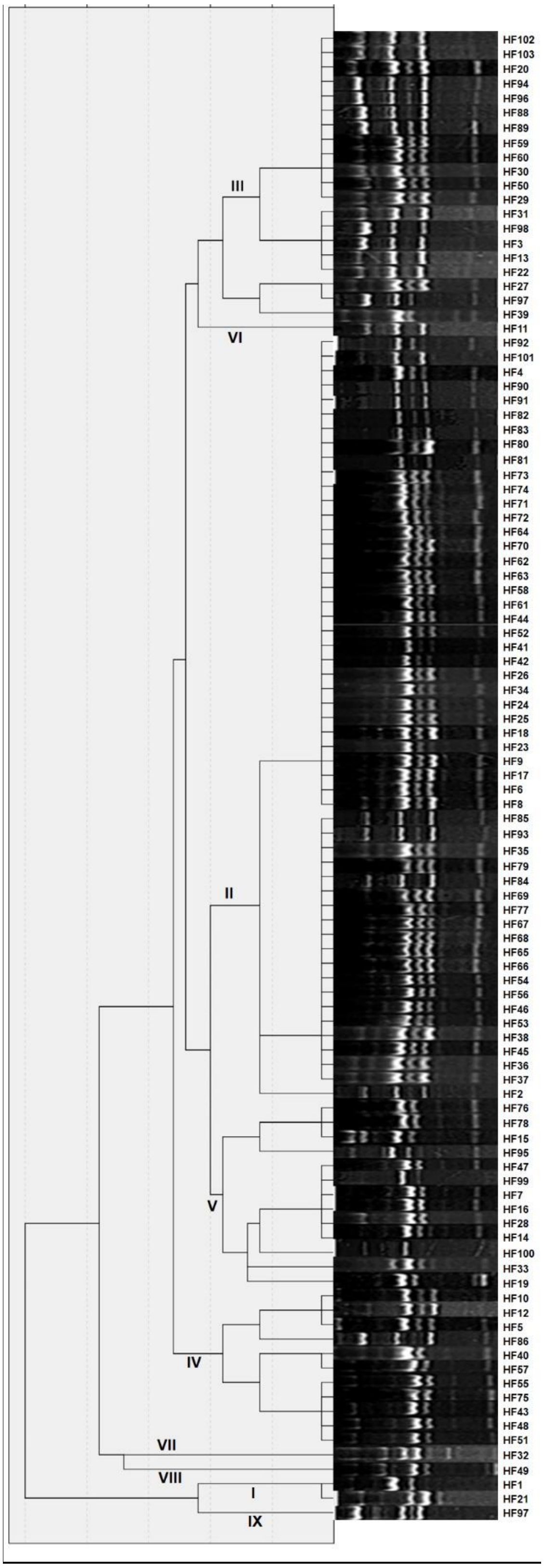

Figure S1 ERIC-PCR fingerprinting and cluster dendrogram of 103 clinical *Acinetobacter baumannii* strains. The roman numerals (I-IX) indicate the different clades identified from the fingerprinting.

FIG. S2

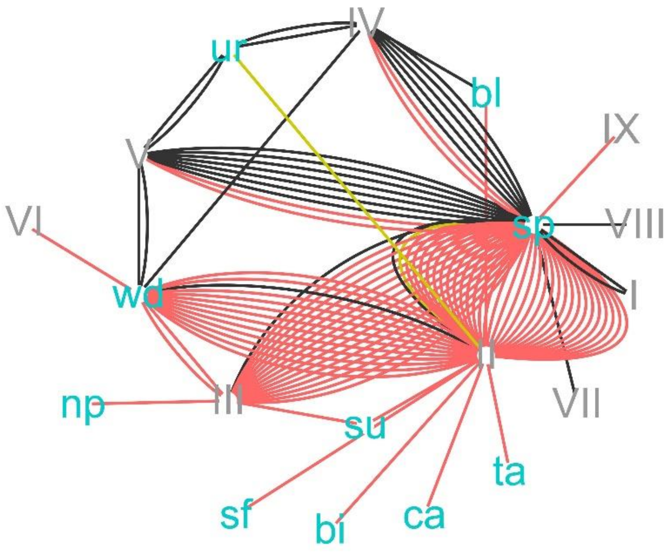

Figure S2 Network between bacterial source and genotypes of carbapenem-resistant *Acinetobacter baumannii* strains. The roman numerals (I-IX) indicate the different clades of strains identified from the ERIC-PCR-dependent fingerprints. Sample source: bi, bile; bl, blood; ca, venous catheter; np, nasopharynx; sf, cerebrospinal fluid; sp, sputum; su, subcutaneous transudation; ta, trachea cannula; ur, urine; wd, wound. Black line, sensitive *A. baumannii*; Orange line, carbapenem-intermediate *A. baumannii*; Red line, carbapenem-resistant *A. baumannii*.

FIG. S3

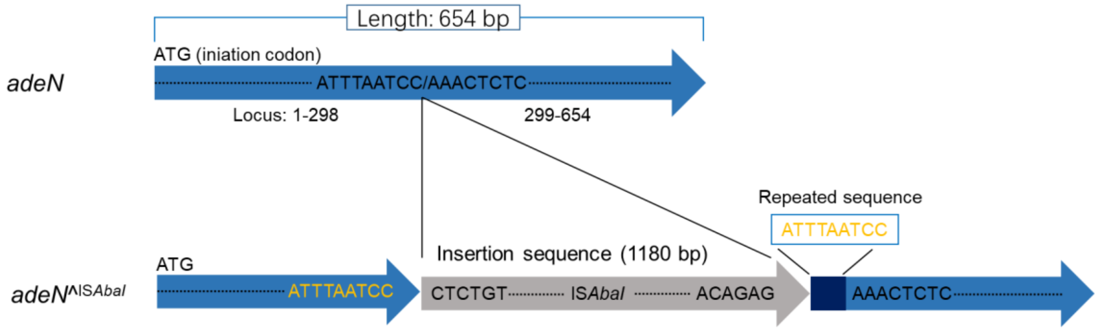

Figure S3 Typical structure of *adeN* with IS*Aba1* insertion in *Acinetobacter baumannii*. Blue arrow refers to sequence of *adeN* gene, gray arrow refers to sequence of IS*Aba1*. Dotted lines refer to omitted bases in sequence.

FIG. S4

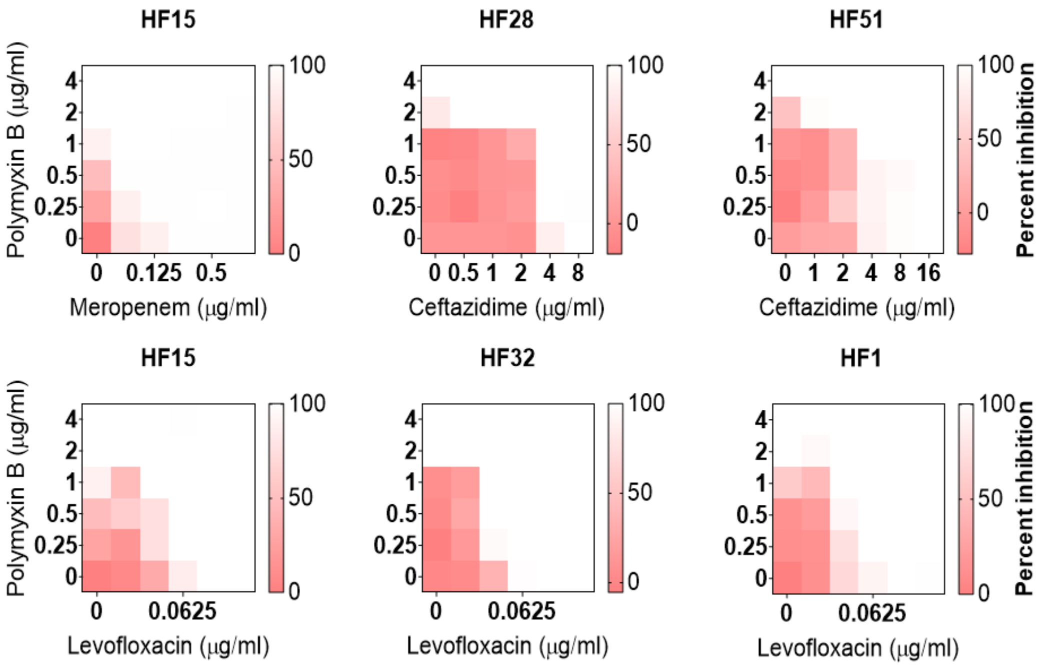

Figure S4 Heat map of combined antimicrobial effect of polymyxin B in combination with meropenem, levofloxacin, and caftazidime against representative strains of *Acinetobacter baumannii*. All data were presented as means of three biological replicates.

REFERENCE:

1. Jia W, Li C, Zhang H, Li G, Liu X, Wei J. 2015. Prevalence of Genes of OXA-23 Carbapenemase and AdeABC Efflux Pump Associated with Multidrug Resistance of *Acinetobacter baumannii* Isolates in the ICU of a Comprehensive Hospital of Northwestern China. Int J Environ Res Public Health 12:10079-92.
2. Coyne S, Guigon G, Courvalin P, Perichon B. 2010. Screening and quantification of the expression of antibiotic resistance genes in *Acinetobacter baumannii* with a microarray. Antimicrob Agents Chemother 54:333-40.

3. Smith M, Gianoulis T, Pukatzki S, Mekalanos J, Ornston L, Gerstein M, Snyder M. 2007. New insights into *Acinetobacter baumannii* pathogenesis revealed by high-density pyrosequencing and transposon mutagenesis. Genes & development 21:601-14.
4. He X, Lu F, Yuan F, Jiang D, Zhao P, Zhu J, Cheng H, Cao J, Lu G. 2015. Biofilm Formation Caused by Clinical *Acinetobacter baumannii* Isolates Is Associated with Overexpression of the AdeFGH Efflux Pump. Antimicrob Agents Chemother 59:4817-4825.
5. Lopez M, Mayer C, Fernandez-Garcia L, Blasco L, Muras A, Ruiz FM, Bou G, Otero A, Tomas M, Geih G. 2017. Quorum sensing network in clinical strains of *A. baumannii*: AidA is a new quorum quenching enzyme. PLoS One 12:e0174454.
6. Poirel L, Figueiredo S, Cattoir V, Carattoli A, Nordmann P. 2008. *Acinetobacter radioresistens* as a silent source of carbapenem resistance for *Acinetobacter* spp. Antimicrob Agents Chemother 52:1252-6.
